# Improving medical research in the United Kingdom

**DOI:** 10.1186/s13104-022-06050-y

**Published:** 2022-05-13

**Authors:** Stephen H. Bradley, Nicholas J. DeVito, Kelly E. Lloyd, Patricia Logullo, Jessica E. Butler

**Affiliations:** 1 Leeds Institute of Health Sciences, University of Leeds, Worsley Building, Leeds, LS2 9JT UK; 2 The DataLab and Centre for Evidence Based Medicine, Nuffield Department of Primary Care Health Sciences, New Radcliffe House, 2nd floor, Radcliffe Observatory Quarter,Woodstock Road, Oxford, OX2 6GG UK; 3 UK EQUATOR Centre, Centre for Statistics in Medicine, NDORMS, University of Oxford, Windmill Road, Oxford, OX3 7LD UK; 4 Centre for Health Data Science, University of Aberdeen, Aberdeen, AB25 2ZD UK

**Keywords:** Reproducibility, Research integrity, Research quality, Research waste, Medical research, Reporting biases

## Abstract

Poor quality medical research causes serious harms by misleading healthcare professionals and policymakers, decreasing trust in science and medicine, and wasting public funds. Here we outline underlying problems including insufficient transparency, dysfunctional incentives, and reporting biases. We make the following recommendations to address these problems: Journals and funders should ensure authors fulfil their obligation to share detailed study protocols, analytical code, and (as far as possible) research data. Funders and journals should incentivise uptake of registered reports and establish funding pathways which integrate evaluation of funding proposals with initial peer review of registered reports. A mandatory national register of interests for all those who are involved in medical research in the UK should be established, with an expectation that individuals maintain the accuracy of their declarations and regularly update them. Funders and institutions should stop using metrics such as citations and journal’s impact factor to assess research and researchers and instead evaluate based on quality, reproducibility, and societal value. Employers and non-academic training programmes for health professionals (clinicians hired for patient care, not to do research) should not select based on number of research publications. Promotions based on publication should be restricted to those hired to do research.

## Introduction

The UK invests substantial sums in medical research and the international reputation of the sector is vital to the government’s industrial strategy [1]. Unfortunately, systemic problems undermine the rigour of medical research and lead to costly research waste [2].

In this commentary, we propose straightforward measures to reduce waste in medical research that will safeguard investments and ensure the UK remains a productive setting for researchers committed to genuine scientific discovery.

## Main text

### What are the causes of the reproducibility crisis in medical research?

While fraud is an important problem that leads to non-reproducible research, many of the issues that undermine reproducibility do not involve deliberate misconduct [3, 4]. Here, we concentrate on identifying and suggesting remedies to the systemic threats to research rigour and reproducibility.

### Lack of transparency for methods and data

Although most UK medical research is funded through taxation and charitable donations, there are remarkably few requirements placed on researchers to share data, adequately describe methods, or provide their full results. Understanding exactly how studies were carried out requires full transparency of study methods and analytic code (where applicable) to verify results and conclusions, ideally with full access to the data. Without providing this information, the validity of published results can only be taken on trust.

Some journals now have policies requiring authors to make their data, analytic code, and protocols available. In practice, these requirements are routinely ignored [5–7]. Considerations of patient privacy or commercial confidentiality are not insurmountable barriers to adequate transparency. Research participants should be offered the opportunity to consent to the sharing of their data, or data can be de-identified and provided via third-party, securely-managed access. The OpenSafely project has provided a successful model throughout the COVID-19 pandemic for safe and secure access to UK patient records for emergency epidemiological research [8]. While individual electronic health records cannot be shared in this instance, the OpenSafely project has made transparency into data access, code, and analysis plans central to the project. The Vivli platform provides another model for securely sharing data collected from clinical research studies and numerous platforms exist for sharing non-sensitive data [9].

Consistent and mandatory sharing of data, code, and protocols via journals is one route to improved transparency. However, there is a strong case that all study documentation for publicly funded research should be reported in the public realm at study completion (regardless of article publication status). The establishment of a single national repository would provide a much more complete and useful record of experiments, as well as reduce duplication in reporting requirements that researchers currently face. The Health Research Authority has recently announced plans for all clinical trials to be automatically registered following ethical approval, in partnership with the ISRCTN registry [10]. This infrastructure could form the nucleus of a more ambitious research repository, hosting comprehensive documentation for all study types.

**Proposed measure 1** Journals and funders should ensure authors fulfil their obligation to share study protocols and, as far as possible, analytical code and research data regardless of the study’s ultimate publication status in a journal.

### Reporting and publication biases

It is well established that results which are ‘positive’ or novel are much more likely to be published [11] leading to a distorted public record. One study found that 96% of research findings were ‘statistically significant’, a mathematical impossibility [12]. Where unpublished results are taken into account, the evidence of benefit for interventions may diminish or disappear altogether [11, 13].

We can monitor whether clinical trials have been published because of legal and ethical requirements that clinical trials are registered prior to commencement [14]. However, clinical trials are a minority of all medical studies. For other study types, including observational studies that have informed healthcare decision-making throughout the coronavirus pandemic, there is little expectation that researchers pre-register their analysis plans and hypotheses. This makes it impossible to monitor what research is planned, if research plans have been followed, if all results are published, and if interpretation involves unscientific “spin” (Table) [15]. Pre-registering study plans for any research type is now straightforward and free to researchers [16, 17]. Requiring pre-registration for medical research can and should be used to promote transparency and accountability.

Although we believe pre-registration enhances transparency and should be more widely used, even when research is pre-registered, it is common to find unacknowledged deviations between the proposed research and the published manuscript [6]. While registration makes these deviations detectable, detection is onerous and journals often fail to take action when alerted to these issues [6]. Related ‘questionable research practices’, like manipulating analyses to generate statistically significant results (‘p-hacking’) and amending study hypotheses retrospectively to suit the results found (Hypothesising After the Result is Known, or ‘HARKing’) can lead to biased findings when research is not pre-registered or the registrations are not checked against final reports [18].

Registered reports are a publishing format with two methods for preventing these poor research practices. In a registered report, reviewers assess methods *before* data collection begins. When the researchers and reviewers agree that the design is appropriate, the researchers are given in-principle approval for publication, regardless of the study findings, as long as the proposed methodology is adhered to [19].

Promoting publishing via registered reports addresses both outcome reporting and publication biases for almost all types of research [19]. Since journals decide on publication based on the methods *before* the results are known, researchers have less incentive to strain to produce eye-catching or positive results. Instead, methodologically sound research enters the record without bias regarding what those results might say. Early evidence suggests that registered reports do improve the quality and rigor of proposed study designs [20, 21]. Unfortunately, medicine lags behind other disciplines like psychology in adopting registered reports, with just over 1% of medical journals offering the format in 2020 [22].

Funders should either incentivise publication via registered report, which will increase their uptake by more journals, or establish their own publication platforms which prioritise the registered report format. The NIHR and Wellcome Trust have both established open and peer-reviewed publication platforms for their funded research [23, 24]. Novel funding pathways are demonstrating that it is possible to integrate peer reviews for registered reports in grant applications which could improve the efficiency of academic discovery and dissemination [25, 26].

**Proposed measure 2** Funders and medical journals should incentivise the uptake of registered reports.

### Lack of transparency on conflicts of interest

Researchers potential conflicts of interest are difficult to ascertain. Conflict of interest statements in publications are brief and often omit important conflicts, even major financial conflicts like sources of funding or relationships with industry [27]. The RetractionWatch project has logged numerous instances of problematic findings, and eventual retractions of articles, due to important undisclosed conflicts of interest [28]. In the UK, there are currently voluntary registers for those who have received payments from the pharmaceutical industry and for doctors [29, 30]. However, because there is little incentive for individuals to make such declarations, these registers are greatly underutilised. Only 0.002% of doctors’ registered with the General Medical Council (GMC) were listed on the doctors’ voluntary register in 2020 [31]. Patients, the public and other scientists are entitled to be able to check whether a researcher has a conflict of interest and to understand what potential biases might impact a study.

There have been calls for the GMC to curate a registry of doctors’ interests [32, 33]. While such a register would mark an important advance in transparency, it would not cover non-medically qualified researchers. Therefore the UK’s research regulator, the Health Research Authority (HRA), along with the GMC, could be tasked with creating a central register, similar to the US OpenPayments database, to index all medical researchers’ interests using the unique identity numbers (ORCiD) which are already required by institutions and funders [34, 35] Expectations of accurate and up-to-date declarations could be encouraged by employers during researchers’ appraisals, although a legal mandate for regulators to ask for such declarations may require legislation [36].

**Proposed measure 3** a mandatory national register of interests for all those who are involved in medical research in the UK should be established, with an expectation that individuals maintain the accuracy of their declarations.

### Dysfunctional incentives and research culture

Decisions about who to hire, fund and promote in academia are often informed using reductive, simple metrics such as citations, the journal impact factor of publications or grant income [37, 38]. Such metrics perversely incentivise researchers to generate a high quantity of publications which are perceived to be exciting or newsworthy, rather than prioritising high quality reproducible research that actually benefits patients and the public. Practices that support high quality research by improving transparency and reducing bias, such as registering studies and publishing all results, are not typically used to appraise performance in academia [39]. Initiatives which seek to mitigate dysfunctional incentives and promote practices that are conducive to reproducible research are becoming more widely established throughout the UK. These include the UK Reproducibility Network (UKRN), the San Francisco Declaration on Research Assessment (DORA), Résumé for Researchers, and the Concordat to Support the Career Development of Researchers [40–44]. Such efforts should be supported and expanded.

**Proposed measure 4** funders and institutions should stop using metrics such as citations and journal’s impact factor to assess research and instead evaluate based on quality, reproducibility and societal value.

Some clinical professionals are incentivised to undertake research because publication is used as a selection criteria for training programmes and for professional promotion [45]. Such dysfunctional incentives promote research that is undertaken solely for career advancement, by individuals who may lack methodological expertise and commitment to produce high-quality reproducible research. There is no persuasive evidence that authoring scientific publications improves the clinical performance of healthcare professionals. Instead we should aspire to create a system of medical research that produces “less research, better research, and research done for the right reasons” [46].

**Proposed measure 5** employers and training programmes for health professionals should remove incentives to publish from their selection procedures.

### Outlook

In this commentary we have set out recommendations to improve the transparency and reproducibility of medical research. Not all of these would be easily implemented, and further evidence is needed to articulate their value and best practice. Moreover sincere engagement from funders, government, journals, researchers, and their employing institutions is required. Elsewhere, we highlighted three actions that warrant prioritisation as readily implementable measures with high potential for impact [22, 47]. These are:

1. Mandatory registration of interests for all people and institutions who conduct and publish health research on a single on-line platform accessible to all;
2. Prioritisation by journals and funders of publication of research via Registered Reports; and.
3. Public pre-registration before data collection of the study design of all publicly-funded medical research, along with protocols, analytic code and, where possible, research data and results.

Table 1 outlines how these actions, along with other proposed measures outlined in this commentary could address some of the problems in medical research.

**Table 1:** Problems in medical research and how they can be mitigated by authors’ proposed strategy

Problem	Problem description	Relevant proposed solutions	How the proposed solutions address the problem
Publication bias	Results deemed ‘negative’ or ‘uninteresting’ are not published	Registered reports	Study accepted for publication based on methods, not results
		Research registry	Study results and documents made available, regardless of article publication status
Misaligned incentives	Researchers are rewarded based on quantity of publications and journal-based metrics rather than on the quality of methods and processes used in research, and healthcare professionals are encouraged to author scientific publications in order to advance in clinical careers	Cease the use of reductionistic metrics like journal impact factor, H-index, and publication counts in assessing researchers. Cease the use of publications in selection criteria for health professionals	Researchers incentivised to focus more on methodological rigour and reproducibility of their research. Healthcare professionals who are not interested in research are not incentivised to publish research
HARKing	Researchers generate hypotheses after results are known—allowing publication of “false positive” findings that are the result of noise in the data rather than true findings	Registered reports, research registry	Hypotheses and aims are agreed prior to undertaking research. Any further post hoc analyses are declared as such
P-hacking	Researchers test many possible hypotheses until one is significant by chance alone, allowing publication of “false positive” findings that are the result of noise in the data rather than true findings	Registered reports	Analyses methods evaluated and approved prior to generation of results
		Research registry	Analysis plans and code available to peers for scrutiny
Outcome switching	Researchers do not report all pre-registered outcomes, or switch primary and secondary outcomes, to highlight results that may be ‘noise’ in the data rather than true findings	Registered reports	Outcomes of interest declared in public prior to undertaking research
		Research registry	Protocols and analysis plans made available to peers for scrutiny
		National register of interests	Conflicting interests which could engender bias made known to public and peers
Spin	Misrepresentation of study results, regardless of motive, that overemphasises the beneficial effects of the intervention and overstates safety compared with that shown by the results [48]	Registered reports	Reduced incentive to ‘spin’ to obtain publication
		Research registry	Study documentation available to allow greater scrutiny of researchers’ claims
		National register of interests	Information on possible conflicts of interest allows peers to judge if researchers have vested interest in applying spin to study
Undisclosed conflicts of interest	Researchers may have vested interest in obtaining certain outcome in their results	National register of interests	Researchers compelled to made comprehensive statement of their pecuniary interests, gifts and hospitality received and non-financial interests
Insufficient methodological details reported and other causes of non-replicable research	Results that cannot be evaluated, either because of insufficient information to reproduce methods or because of biases in original study produced significant results by chance rather than by detecting a true signal	Research registry reporting guidelines	Adequate study documentation made available and in enough detail such that the study can be reproduced or analyses repeated

There is growing acknowledgement that systemic problems pervade health research. But as long as we fail to take action, medicine’s reproducibility crisis will persist. The recommendations in this commentary aim to advance beyond identification of the problems to meaningful, achievable reform.

## Data Availability

Not applicable.

## Abbreviations

ISRCTN: International standard randomised controlled trial number
UKRN: United Kingdom reproducibility network
